# A Novel Strategy to Identify Haematology Patients at High Risk of Developing Aspergillosis

**DOI:** 10.3389/fimmu.2021.780160

**Published:** 2021-12-16

**Authors:** James S. Griffiths, P. Lewis White, Aiysha Thompson, Diogo M. da Fonseca, Robert J. Pickering, Wendy Ingram, Keith Wilson, Rosemary Barnes, Philip R. Taylor, Selinda J. Orr

**Affiliations:** ^1^ Division of Infection and Immunity and Systems Immunity Research Institute, Cardiff University School of Medicine, Cardiff, United Kingdom; ^2^ Centre for Host-Microbiome Interactions, Faculty of Dentistry, Oral and Craniofacial Sciences, King’s College London, London, United Kingdom; ^3^ Public Health Wales Microbiology Cardiff, University Hospital of Wales (UHW), Cardiff, United Kingdom; ^4^ United Kingdom (UK) Dementia Research Institute at Cardiff, Cardiff, United Kingdom; ^5^ Wellcome-Wolfson Institute for Experimental Medicine, School of Medicine, Dentistry and Biomedical Science, Queen’s University Belfast, Belfast, United Kingdom; ^6^ The Institute of Cancer Research, London, United Kingdom; ^7^ University Hospital of Wales, Cardiff, United Kingdom

**Keywords:** CLR, *Aspergillus*, aspergillosis, fungal immunology, host-pathogen interactions

## Abstract

Invasive Aspergillosis (IA), typically caused by the fungus *Aspergillus fumigatus*, is a leading cause of morbidity and mortality in immunocompromised patients. IA remains a significant burden in haematology patients, despite improvements in the diagnosis and treatment of *Aspergillus* infection. Diagnosing IA is challenging, requiring multiple factors to classify patients into possible, probable and proven IA cohorts. Given the low incidence of IA, using negative results as exclusion criteria is optimal. However, frequent false positives and severe IA mortality rates in haematology patients have led to the empirical use of toxic, drug-interactive and often ineffective anti-fungal therapeutics. Improvements in IA diagnosis are needed to reduce unnecessary anti-fungal therapy. Early IA diagnosis is vital for positive patient outcomes; therefore, a pre-emptive approach is required. In this study, we examined the sequence and expression of four C-type Lectin-like receptors (Dectin-1, Dectin-2, Mincle, Mcl) from 42 haematology patients and investigated each patient’s anti-*Aspergillus* immune response (IL-6, TNF). Correlation analysis revealed novel IA disease risk factors which we used to develop a pre-emptive patient stratification protocol to identify haematopoietic stem cell transplant patients at high and low risk of developing IA. This stratification protocol has the potential to enhance the identification of high-risk patients whilst reducing unnecessary treatment, minimizing the development of anti-fungal resistance, and prioritising primary disease treatment for low-risk patients.

## Introduction

Invasive aspergillosis (IA) has become a leading cause of death among immunocompromised patients (1–3). The disease, mainly caused by *Aspergillus fumigatus*, affects ~ 10% of allogeneic stem cell transplant (SCT) patients and ~6% of acute myeloid leukaemia (AML) patients. IA is associated with unacceptably high mortality rates ranging from 30-40% in AML patients and 50-90% in SCT patients (4–9). IA is particularly prevalent in patients with haematologic malignancies. This is attributed to the profound immune suppression and neutropenia brought about by the extensive therapeutic use of cytotoxic chemotherapies, radiation therapy, requirement for SCT and the use of corticosteroids and immunomodulatory therapies (10). Whilst the diagnosis and treatment of A*spergillus* infections is improving, severe IA-associated morbidity and mortality in haematology patients has led to the widespread, empirical use of anti-fungal prophylaxis in this patient group (11, 12). Current anti-fungal therapeutics can be ineffective, encounter resistance, are poorly tolerated and highly drug interactive, often impacting patient’s primary cancer therapies (13).

Improvements in IA diagnostics and the identification of novel risk factors are required to stratify patients prior to infection and enhance the early diagnosis of IA, thus providing a personalised medicine approach that better targets anti-fungal therapy. Assessing a patient’s IA risk and then accurately and rapidly diagnosing IA remains challenging. Initially, a patient’s underlying clinical conditions will govern IA risk, with patients being grouped into low, medium and high risk cohorts (14). Patients are classified with possible IA, probable IA and proven IA through the presence of host factors, and clinical and microbiological evidence. However, proven IA is often only confirmed post-mortem. A range of routine mycological investigations are available, both novel (*Aspergillus* PCR, galactomannan enzyme immunoassay, 1-3-β-D-glucan detection) and conventional (culture and microscopy), and are usually combined with radiology typical of IA. However, the availability of these tests varies considerably, they can produce false positive results, some lack sensitivity, are not always *Aspergillus* specific, are impacted by anti-fungal therapies, and can require invasive sampling (15, 16). Therefore, an improved strategy to promote the rapid and accurate diagnosis of IA is required.

The accurate, early diagnosis of *Aspergillus* infection is vital for positive patient prognosis (17). Whilst the development of more sensitive assays has improved IA diagnosis, the identification of novel risk factors that increase IA susceptibility is central to promoting a personalised medicine approach to anti-fungal investigations and treatment. Multiple risk-factors for IA have been identified, many of which are associated with the haematology patient population. Neutropenia was the first described IA risk factor and is frequently encountered in haematology patients following primary disease treatment (18). Since then, allogeneic stem cell transplantation, graft versus host disease and respiratory infection have been associated with increased IA susceptibility (19–21). Whilst the identification of these risk factors is associated with a higher prevalence of IA in haematology patients, they do not permit a pre-emptive personalised medicine approach as these risk factors are common and often unavoidable.

The identification of genetic risk factors that increase fungal susceptibility has promoted a pre-emptive approach to determining haematology patient IA risk. These genetic risk factors can be routinely screened for and used to inform a patient’s anti-fungal investigations and therapies (22). Genetic mutations that increase fungal susceptibility have been found in innate and adaptive anti-fungal immune components. One of the most important facets of anti-fungal immunity is the C-type Lectin-like Receptor (CLR) family. CLRs are pattern recognition receptors that recognise pathogen associated molecular patterns, specifically carbohydrate structures present in the fungal cell wall (23). CLRs such as Dectin-1 and Dectin-2 are essential for anti-fungal immunity and recognise most, if not all, fungal species that cause human disease (24, 25). Deficiencies in these CLRs have been associated with increased susceptibility to invasive fungal infection (26–28). Upon fungal recognition CLRs induce intracellular signalling and drive the production of cytokines and chemokines, phagocytosis, and respiratory burst (26, 29). Additionally, CLRs have been shown to mediate protective Th1 and Th17 immunity during systemic and mucosal fungal infection (30, 31). Directly determining an individual’s functional response to fungi through peripheral blood mononuclear cell (PBMC) functional assays has the capacity to identify immune deficiencies associated with a wide array of genetic mutations (including novel mutations) that predispose the patient to IA.

Stratifying patients according to their IA risk prior to their primary treatment and immune suppression would permit a personalised medicine approach and reduce the empirical prophylactic use of anti-fungal therapies. In this study we investigated CLR status and anti-*Aspergillus* immune response for a small cohort of haematology patients. Samples were collected from 42 AML and SCT patients. Each patient was screened for exonic CLR (Dectin-1, Dectin-2, Mcl, Mincle) mutations and their mRNA expression level was quantified. PBMCs were isolated and functional assays were performed to determine each patient’s LPS- and *Aspergillus*-induced cytokine (IL-6/TNF) response. Each patient’s CLR status and functional response results were then associated with the incidence of IA and these results were used to identify new IA risk factors. Our research describes a novel strategy that permits the pre-emptive stratification of haematology patients according to their IA susceptibility and drives a personalised medicine approach to their anti-fungal therapy.

## Materials and Methods

### Patient Study Information

The clinical research project was undertaken with sponsorship from Cardiff University, support from the University of Wales teaching hospital haematology and Public Health Wales Microbiology departments, and ethical approval from Health and Care Research Wales (NISCHRC CRC 1351-14). REC reference 14/WA/1119 and IRAS project ID 151136. Written informed consent was obtained from all patients in the study. Whole blood samples were collected from 42 acute-myeloid leukaemia and stem cell transplant patients upon admission to hospital for their primary disease treatment. Fungal disease investigations were undertaken according to local health board guidelines. Of the 42 patients enrolled in the study, 9 developed probable IA according to the European Organization for Research and Treatment of Cancer/Invasive Fungal Infections Cooperative Group and the National Institute of Allergy and Infectious Diseases Mycoses Study Group (EORTC/MSG) consensus definitions (32). Patient’s primary disease, anti-fungal treatment and survival are included in **Table 1**. This study tracked 42 patient’s fungal disease status and survival from 7^th^ September 2015 until 9^th^ April 2018. Each patient was anonymised and assigned a number which was consistent throughout the study. The results in this study show patients 1 to 43, as the samples from patient 18 were not processed. Not all assays were undertaken for each patient and statistical analysis was only completed on patient results that met all investigated parameters. Therefore, patient numbers throughout results and analysis vary.

**Table 1:** Patient information including primary demographics, clinical parameters, mortality, and anti-fungal prophylaxis for no evidence of fungal disease (NEF) and invasive aspergillosis (IA) patients.

Parameter		NEF (n=33)	IA (n=9)
Patient Demographics	Female	13	5
	Male	20	4
	Age Median (Range)	59 (21 to 76)	52 (23 to 72)
Clinical parameters	AML	15	4
	SCT	18	5
	Neutropenia* (% of total)	27 (82%)	7 (78%)
Mortality	Total	11	4
	AML	5	1
	SCT	6	3
Anti-fungal prophylaxis	Fluconazole	20	7
	Posaconazole	1	1
	Voriconazole	0	2

*Defined as 10 consecutive days of <0.5x10^9^/L neutrophils in whole blood within one month prior to a diagnosis of IA or across the duration of the study.

### Samples Collected

17.5ml Whole blood was collected from each patient into EDTA tubes (BD) and 2ml whole blood from was collected from each patient into PAXgene blood RNA tubes (Preanalytix) following initial admission to hospital. The 17.5ml whole blood sample was immediately processed for use in the functional assays. The 2ml whole blood in PAXgene blood RNA tubes was stored according to manufacturer’s instructions for up to 6 months before being processed in batches.

### RNA Isolation

Patient RNA samples were processed in batches of 8 and were not stored for longer than 6 months at -80°C. PAXgene blood RNA tubes were equilibrated at room temperature for 2 h following storage at -80°C before RNA was extracted from the whole blood within the PAXgene blood RNA tubes according to the manufacturer’s instructions. The optional DNA digestion step was completed for every patient sample. Typically, ≥3μg RNA was obtained from each patient sample and quantified by nanodrop (Thermo Scientific). The integrity of extracted RNA was confirmed by running patient RNA samples on a 1% agarose gel and visualising the 18S and 28S rRNA bands.

### cDNA Generation

Patient RNA was used to generate cDNA using a Quantitect Reverse Transcription Kit (Qiagen). Typically, 2000ng of cDNA was generated for each patient at a final concentration of 100ng/µl for immediate use in Real-Time qPCR. Successful cDNA generation was confirmed by running patient RNA and cDNA samples on a 1% agarose gel. The absence of the 18S and 28S rRNA bands in the cDNA sample indicated successful cDNA generation.

### CLR Gene Expression and Sequencing

Gene expression levels of the CLRs Dectin-1 (*CLEC7A*), Dectin-2 (*CLEC6A*), Mcl (*CLEC4D*) and Mincle (*CLEC4E*) was determined by Real-Time qPCR using the Taqman qPCR Mastermix (Thermo Scientific) and CLR gene-specific primer and probe sets (Thermo Scientific) detailed in **Supplementary Table 1**. 100ng of patient cDNA was used in each CLR qPCR reaction. Gene expression normalization was performed against *HPRT1*. Patient CLR exon sequences were determined by PCR amplification of each CLR gene from patient cDNA using the primers detailed in **Supplementary Table 1**. CLR DNA was purified from PCR reaction mixtures using a PCR purification kit (Qiagen) and sent for sequencing at GATC Biotech.

### PBMC Culture

17.5ml blood collected into EDTA tubes was added to 50ml conical tubes on top of an equal volume of Ficoll Plus (Sigma) and centrifuged at 400 x g for 30 min with the centrifuge brake reduced to its lowest setting. After centrifugation the layer of PBMCs was removed, washed once with PBS (Life Technologies) and then three times with RPMI 1640 (Life Technologies) before being counted and resuspended in RPMI 1640 supplemented with 10% FBS (Life Technologies), 2% Human Serum (Sigma), 10mM L-glutamine (Life Technologies), 10mM Sodium Pyruvate (Life Technologies) and 100µg/ml Gentamycin (Life Technologies). 100µl of 5x10^6^/ml PBMCs were added to each required well on a 96-well plate (Thermofisher) and rested at 37°C for 4 h prior to the functional assay.

### *Aspergillus* Culture

*Aspergillus fumigatus* (isolate 13073) was cultured on potato dextrose agar (Sigma) for 7 days before resting conidia (RC) were harvested by vigorous washing with PBS 0.05% Tween 20 (Sigma). Harvested RC were counted and stored at 4°C in PBS 0.05% Tween 20 for a maximum of 6 weeks before a new culture was started. For functional assays, RC were grown in RMPI 1640 supplemented with 0.2mg/ml Polymixin B (Sigma) at 37°C 5% CO_2_ for 6 hours to generate swollen conidia (SC). *Aspergillus fumigatus* SC were counted and resuspended at 5x10^6^ SC/ml for use in functional assays.

### Cytokine Assay

100µl of 5x10^6^/ml PBMCs were stimulated with 100µl of 1µg/ml LPS (Sigma) or 100µl of 5x10^6^ *Aspergillus fumigatus* swollen conidia/ml for 24 h at 37°C 5% CO_2_. An unstimulated media-only control was included. After 24 h, supernatant was removed from each well and TNF and IL-6 levels in the supernatants were quantified by ELISA (eBioscience).

### Statistical Analysis

Significance was determined using contingency multivariate statistical analysis with Fisher’s exact test. If two variables were analysed *p=0.05, **p=0.005. Where more than two variables were analysed Bonferroni’s correction was applied and adjusted *p* values are described in the Figure legend.

## Results

### Haematology Patients Display Varied CLR Expression Levels and Mutations

**Figure 1:**
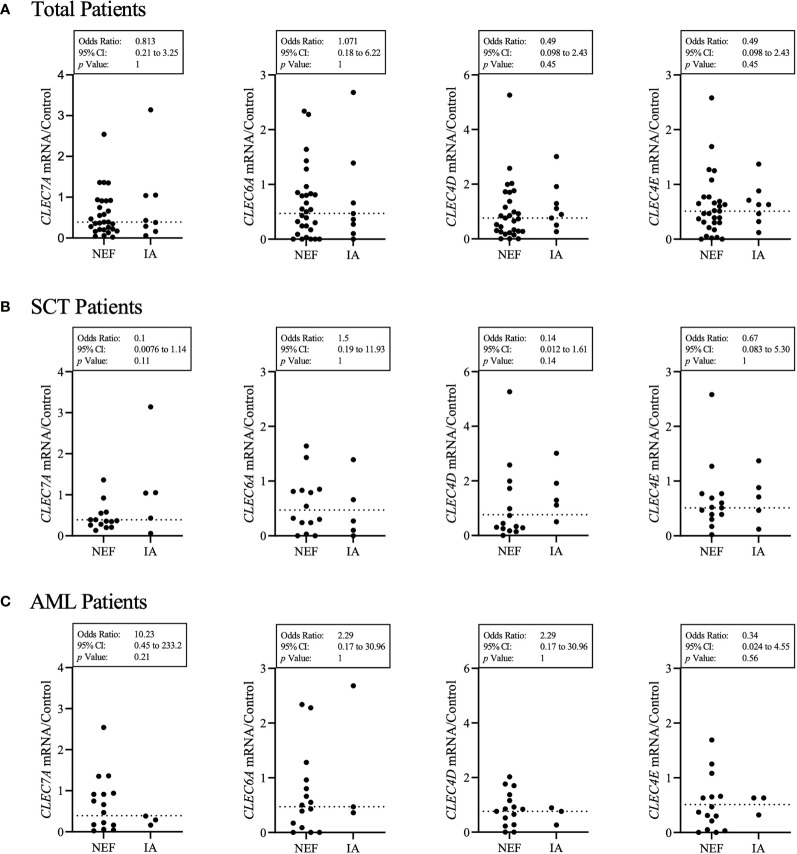
Patient’s CLR status does not clearly identify those susceptible to IA. **(A)** Each patient’s *HPRT1* and CLR gene expression (*CLEC7A* – Dectin-1, *CLEC6A* – Dectin-2, *CLEC4D* – Mcl, *CLEC4E* – Mincle) was quantified by qPCR. Results displayed were calculated using ΔΔCt comparison against HPRT and CLR results from a healthy control sample. The healthy control CLR results were set at a value of 1 for each CLR. The dotted line represents the median for each CLR gene expression from 37 patients. IA represents probable IA. NEF represents no evidence of fungal disease. **(A)** Displays total patient results, **(B)** displays SCT patient results and **(C)** displays AML patient results. Statistical analysis was produced from a contingency multivariate statistical analysis of low CLR expression associated with the incidence of IA. Results described as low were below the median calculated for each CLR from all patient results. Fisher’s exact test was used to identify statistical significance. As two variables were examined, statistical significance was set at *p* < 0.05.

Previous studies have associated SNPs in Dectin-1, Dectin-2 and CARD9 with IA (33–36); therefore, we first aimed to determine the sequence and expression levels of four fungal binding CLRs in our cohort of SCT and AML patients. We screened 42 patients for mutations in the exon coding regions of four CLRs (Dectin-1, Dectin-2, Mincle and Mcl) and identified a Dectin-2 mutation (N170I) (rs1334241354) (34) present in 1 patient and an Mcl mutation (S32G) (rs4304840) present in 17 patients. The Dectin-2 mutation resulted in an early stop codon and loss of the carbohydrate binding region whereas the Mcl mutation only resulted in a single amino acid substitution. We next quantified gene expression of *CLEC7A* (Dectin-1)*, CLEC6A* (Dectin-2)*, CLEC4D* (Mcl) and *CLEC4E* (Mincle) by Real-Time qPCR for each patient (**Figure 1**). Expression levels of the four CLRs varied considerably from patient to patient and no clear association between CLR expression level and the incidence of IA was observed.

### Patient CLR Expression Levels Are Not Associated With IA

In order to investigate whether CLR expression was associated with the incidence of IA, we grouped each patient’s CLR results into high (above the median) or low (below the median) and analysed whether high or low CLR expression was associated with IA incidence (**Figure 1**). Here we found that AML patients who developed IA were 10.2 times more likely to have low Dectin-1 expression. In contrast, SCT patients who developed IA were 7.1 times less likely to have low Mcl expression, and 10 times less likely to have low Dectin-1 expression. However, the overall results associating CLR expression with IA incidence across total, AML and SCT patient groups did not provide significant associations.

### Patient CLR Mutations Are Not Associated With IA

We next determined whether CLR mutations were associated with IA incidence (**Table 2**). We did not find any association between the incidence of the Mcl S32G mutation and the incidence of IA. Patients with the mutation were equally distributed between the IA group and the no evidence of fungal infection (NEF) group. In addition, as low Mcl expression in SCT patients reduced the likelihood of developing IA within the study, we also examined whether the Mcl S32G affected Mcl gene expression and found no association. The single patient with the Dectin-2 N170I mutation did develop IA (34); however, as this mutation was only present in one patient statistical correlation analysis was not appropriate. Overall, we did not observe any significant association between the CLR mutations and expression identified in this study and the incidence of IA.

**Table 2:** Mcl S32G does not affect Mcl gene expression or the incidence of IA.

Patient Group	Parameter	IA	NEF	Odds Ratio	95% CI	*p* Value
Total (42)	Mcl S32G	5/9	12/33	2.19	0.49 to 9.74	0.446
AML (19)	Mcl S32G	2/4	5/15	2	0.21 to 18.7	0.603
SCT (23)	Mcl S32G	3/5	7/18	2.36	0.31 to 17.85	0.618
**Patient Group**	**Parameter**	**Low Mcl Expression**	**High Mcl Expression**	**Odds Ratio**	**95% CI**	***p* Value**
Total (37)	Mcl S32G	11/19	6/18	2.75	0.72 to 10.48	0.192

This data was produced from a contingency multivariate statistical analysis of incidence of Mcl mutant against incidence of IA, and incidence of Mcl mutant against Mcl expression. CLR expression as determined in **Figure 1** was used in this analysis. IA represents probable IA. NEF represents no evidence of fungal disease. Fisher’s exact test was used to identify statistical significance. As two variables were examined, statistical significance was set at p < 0.05.

### Most IA Patients Lack an IL-6/TNF Response to *A. fumigatus*

**Figure 2:**
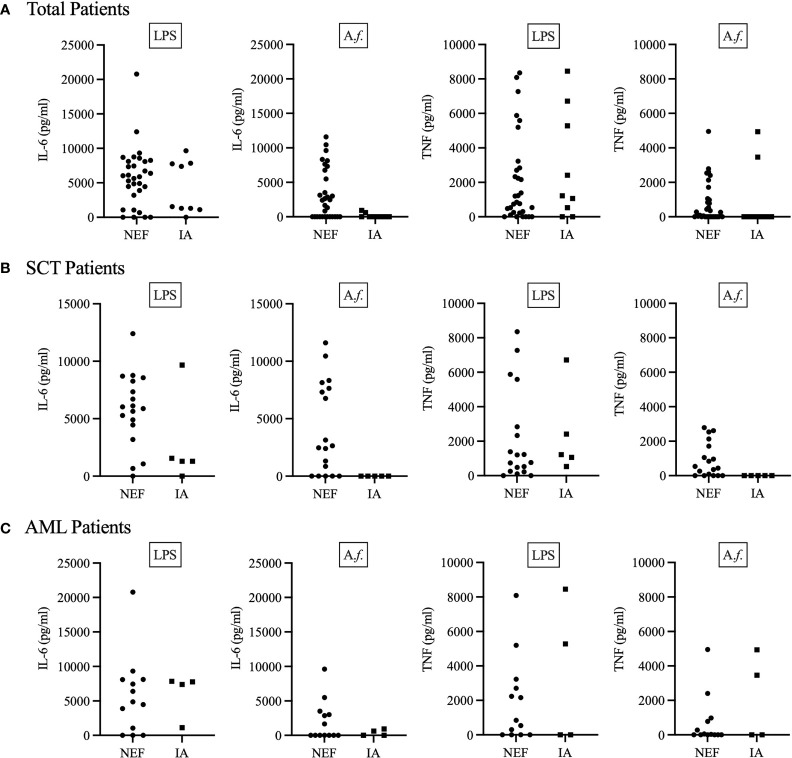
IA patients may lack a specific anti-*Aspergillus* TNF and IL-6 cytokine response. Patient PBMCs were isolated from whole blood and stimulated with 1μg/ml LPS or 5x10^6^ *Aspergillus fumigatus* RC/ml for 24 h. **(A)** Displays total patient results, **(B)** displays SCT patient results and **(C)** displays AML patient results. 24 h after stimulation, supernatant was collected, and the concentration of TNF and IL-6 determined by ELISA. IA represents probable IA. NEF represents no evidence of fungal disease. Statistical analysis for these graphs is presented in **Table 3**. Data from these graphs are the same as in **(A)** from a previous publication from our group (34).

As the cytokines IL-6 and TNF are vital for a protective anti-*Aspergillus* immune response (37, 38), we next decided to investigate each patient’s functional anti-*Aspergillus* immune response. To this end, we isolated patient PBMCs and challenged them with LPS or *Aspergillus fumigatus* SC for 24 h before quantifying the IL-6 and TNF cytokine response (**Figures 2A–C**). Here, our results suggest the majority of patients were able to generate LPS-induced TNF and IL-6; however, fewer patients were able to generate *Aspergillus*-induced TNF and IL-6. Interestingly, whilst all 9 of the IA positive patients produced LPS-induced cytokines, only three IA positive patients (all in the AML cohort) generated *Aspergillus*-induced TNF or IL-6, and only one of these patients produced both cytokines (**Figures 2A, C**). Additionally, only 11 out of 17 AML patients were able to produce any TNF or IL-6 response (**Figure 2C**). This was likely due to AML patient’s highly disrupted haematopoietic compartment and lack of mature myeloid cells. However, for SCT patients our data suggests that IA patients may lack a specific anti-*Aspergillus* response. All 5 IA positive SCT patients produced LPS-induced TNF and 4 of the 5 IA positive SCT patients produced LPS-induced IL-6, whilst none of these IA positive patients produced either cytokine following *Aspergillus* challenge (**Figure 2B**). Similarly, the majority (all but one) of the NEF patients produced LPS-induced TNF and/or IL-6, but in contrast to the IA patients, 13 out of 18 NEF patients generated *Aspergillus*-induced TNF and/or IL-6 (**Figure 2B**). The samples used to generate these results were isolated from patients upon admission to hospital for their primary disease treatment. At the time the assays were performed all IA patients in the study had monocyte counts within the normal range. Therefore, this assay may stratify SCT patients according to their risk of developing IA prior to the patients becoming highly susceptible to invasive fungal disease.

### Lack of IL-6/TNF Production as a Risk Factor for IA in SCT Patients

In order to determine whether a patient’s functional immune response could be used as a risk factor for IA, we associated each patient’s functional response results with the incidence of IA (**Table 3**). Each patient’s functional response results were included as the single parameter no *Aspergillus*-induced TNF or IL-6 response. Here, we identified a significant association between patient’s functional immune response and the incidence of IA. The strongest association was identified in SCT patients who lacked an *Aspergillus*-induced IL-6 response. This factor defined 5 out of 5 SCT patients who developed IA and only 5 out of 18 SCT NEF patients. Patients who fulfilled these criteria were 27 times more likely to develop IA when compared to SCT patients who generated *Aspergillus*-induced IL-6. SCT patients who did not produce an *Aspergillus*-induced TNF response were also significantly more likely to develop IA, with over 21 times higher IA risk within our study. Here, we identify novel IA functional risk factors that could be used to stratify patients according to their IA susceptibility.

**Table 3:** Patients who lack a functional response against *Aspergillus* are more susceptible to IA.

Patient Group	Parameter	IA	NEF	Odds Ratio	95% CI	*p* Value
Total (40)	No *Aspergillus* TNF response	7/9	12/31	5.542	0.98 to 31.25	0.0601
Total (40)	No *Aspergillus* IL-6 response	7/9	12/31	5.542	0.98 to 31.25	0.0601
						
AML (17)	No *Aspergillus* TNF response	2/4	6/13	1.167	0.12 to 11	1
AML (17)	No *Aspergillus* IL-6 response	2/4	7/13	0.857	0.09 to 8.07	1
						
SCT (23)	No *Aspergillus* TNF response	5/5	6/18	21.15	1.06 to 445	*0.0137
SCT (23)	No *Aspergillus* IL-6 response	5/5	5/18	27	1.27 to 575	*0.0075
						

This data was produced from a contingency multivariate statistical analysis of patient PBMC functional assay results as displayed in **Figure 2**. IA represents probable IA. NEF represents no evidence of fungal disease. Fisher’s exact test was used to identify statistical significance. Where two variables were examined, statistical significance was set at *p < 0.05; significant values are highlighted bold.

### Lack of IL-6/TNF Production Is Not Associated With Mortality

Whilst we have described the significant association between *Aspergillus*-induced TNF and IL-6 and the incidence of IA, it was important to determine whether these results were specific for predicting IA or a more general indicator of poor patient prognosis. Therefore, we associated the incidence of IA and patient’s functional immune response results with mortality (**Supplementary Table 2**). Here, we found no association between the incidence of IA or patient’s functional immune response results with the incidence of mortality. These results suggest the TNF and IL-6 functional immune response results are specific risk factors for the incidence of IA within our cohort and not simply identifying patients with a high incidence of mortality.

### Combination Risk Factors and IA

As we found a significant association for patient’s functional immune response and IA, we next analysed multiple risk factors in combination. Patient’s CLR status and functional immune responses were combined and associated with the incidence of IA (**Table 4**). Here, our data shows SCT patients that produced LPS-induced TNF and/or IL-6, lacked *Aspergillus*-induced TNF and/or IL-6 and had high Mcl expression possessed a high risk of IA. Of the 6 patients that fulfilled these parameters, 4 (66%) went on to develop IA. Our data suggests the highest IA risk is present in patients that produced LPS-induced TNF and/or IL-6, lacked *Aspergillus*-induced TNF and/or IL-6 and had high Dectin-1 expression or both high Dectin-1 and high Mcl expression. These parameters stratified 5 patients from the total SCT cohort and 4 developed IA. Interestingly, patient 39 was positive for *Aspergillus* mycology but their radiological investigation did not show evidence of IA; therefore, the patient was not classified as probable IA in accordance with EORTC/MSG guidelines (32). In our study patient 39 had high Mcl and Dectin-1 expression, produced LPS-induced IL-6 and/or TNF and lacked *Aspergillus*-induced IL-6 and/or TNF, suggesting this patient had a high risk of IA. Patient 39 was the only patient stratified into the highest risk cohort that did not have proven IA.

**Table 4:** SCT patients can be stratified according to IA risk using their CLR status and functional immune response results.

Patient group	Risk Factor(s)	Incidence in Patient Cohort	Incidence of IA	Odds Ratio	*p* Value
SCT (23)	No *Aspergillus* TNF response	11/23	5 (45%)	21.15	*0.0137
SCT (23)	No *Aspergillus* IL-6 response	10/23	5 (50%)	27	*0.0075
SCT (22)	LPS TNF/IL-6 response + no *Aspergillus* TNF/IL-6 response	8/22	5 (62.5%)	45.57	**0.0021
					
SCT (19)	High Mcl expression	9/19	4 (44%)	7.2	0.14
SCT (19)	High Dectin-1 expression	8/19	4 (50%)	10	0.11
SCT (18)	High Mcl expression + LPS TNF/IL-6 response + no *Aspergillus* TNF/IL-6 response	6/18	4 (66%)	45	**0.0049
SCT (18)	High Dectin-1 expression + LPS TNF/IL-6 response + no *Aspergillus* TNF/IL-6 response	5/18	4 (80%)	81	**0.0016
SCT (18)	High Dectin-1 expression + High Mcl expression + LPS TNF/IL-6 response + no *Aspergillus* TNF/IL-6 response	5/18	4 (80%)	81	**0.0016
					
AML (17)	No *Aspergillus* TNF response	8/17	2 (25%)	1.167	1
AML (17)	No *Aspergillus* IL-6 response	9/17	2 (22%)	0.857	1
AML (17)	LPS TNF/IL-6 response + no *Aspergillus* TNF/IL-6 response	3/17	1 (33%)	0.545	1
					
AML (18)	Low Dectin-1 expression	9/18	3 (33%)	10.23	0.205

This data was produced from a contingency multivariate statistical analysis of patient CLR status and PBMC functional assay results as displayed in **Figures 1**, **2**. IA represents probable IA. NEF represents no evidence of fungal disease. Fisher’s exact test was used to identify statistical significance. Where two variables were examined, statistical significance was set at *p < 0.05, **p < 0.005; significant values are highlighted bold. Where more than two variables were examined, Bonferroni’s correction was applied; significant values are highlighted bold and italic.

In **Table 4** we also display the incidence of IA within the SCT cohort according to the risk factor parameters used. Here, 5 of 11 (45%) SCT patients with no *Aspergillus*-induced TNF response developed IA, 5 of 10 (50%) SCT patients with no *Aspergillus*-induced IL-6 response developed IA, and 5 of 8 (62.5%) SCT patients with an LPS-induced TNF and/or IL-6 response but lacking an *Aspergillus*-induced TNF and/or IL-6 response developed IA. We next investigated whether the CLR status and functional immune response risk factors could be used to predict the incidence of IA in AML patients within the study. Here, we found no association between AML patients’ functional responses and the incidence of IA. Using low Dectin-1 expression as a risk factor stratified 9 patients from the total AML cohort of which 3 (33%) developed IA but this result was not significant.

### Proposed Pre-Emptive Stratification System for SCT Patients

**Figure 3:**
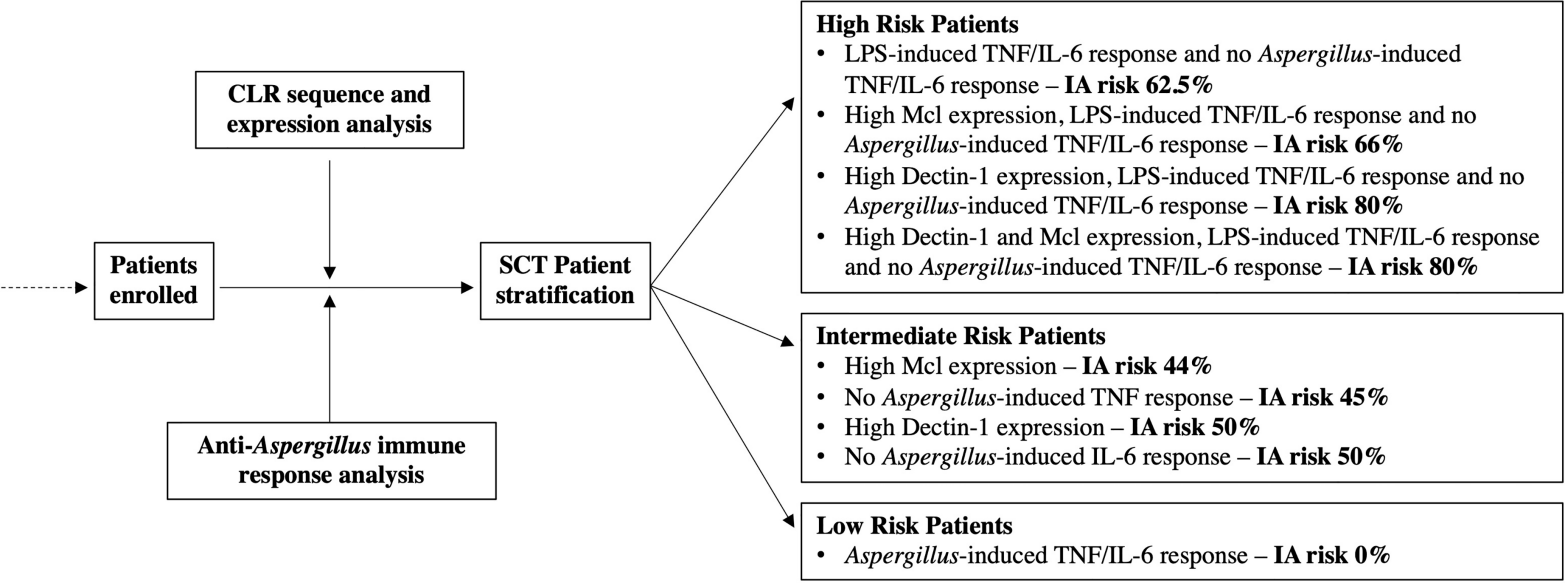
Stratification of SCT patients according to IA susceptibility using risk factors identified in this study. This schematic figure was produced from the results displayed **Figures 1**, **2** and **Table 4**. The proposed strategy utilises the risk factors and incidence of IA from this study and stratifies patients into three groups according to IA susceptibility.

Finally, we used the risk factors and associated IA incidence described for SCT patients to propose a strategy that would enable the pre-emptive stratification of SCT patients according to their IA susceptibility (**Figure 3**). We show in the intermediate- and high-risk groups how the risk factors identified in **Table 4** could be used to predict the incidence of IA within the study. We also demonstrate how the risk factors described in this study could be used as exclusion criteria to stratify patients at low risk of IA. Importantly, none of the SCT patients that produced *Aspergillus*-induced TNF and/or IL-6 developed IA.

In this study our investigations into patient’s CLR status and functional immune response have led to the identification of novel IA risk factors. We have demonstrated how these risk factors may be applied to stratify patients into low, intermediate, and high-risk cohorts. Crucially, the risk factors identified here would enable the pre-emptive stratification of patients and permit a personalised medicine approach to patient’s anti-fungal investigations and treatment.

## Discussion

The aims of this pilot study were to identify novel risk factors that could stratify haematology patients according to their IA susceptibility. The development and incorporation of biomarkers to assist in the pre-emptive management of haematology patients at risk of IA have demonstrated significant utility for excluding disease, but even in the presence of multiple positive results the positive predictive value (post-test probability) for confirming IA is not optimal (15). Whilst there are well described IA risk factors, these are frequently encountered in the haematology patient population and IA-associated mortality remains unacceptably high. Therefore, highly drug-interactive, and sometimes ineffective anti-fungal therapy is often empirically administered at the first sign of refractory infection or prophylactically administered to asymptomatic patients deemed high risk through host factors or clinical intervention. Ultimately, this results in haematology patients receiving frequent fungal clinical investigations and unnecessary anti-fungal therapy. Identifying patients at low or high risk of infection through host biomarker testing prior to that patient becoming immune suppressed and susceptible to IA would target anti-fungal prophylaxis and allow a personalised medicine approach to managing the haematology patient.

### CLR Status and IA

In this study we investigated patient’s CLR status as deficiencies in anti-fungal immune components have previously been associated with IA. The first inherited or acquired genetic condition associated with IA susceptibility was identified in CARD9, a downstream adaptor molecule that transduces CLR signalling. CARD9-deficiency resulted in fungal infections without any immune suppression (35, 36, 39). The Dectin-1 Y238X mutation increases IA susceptibility in haematology patients through reduced fungal recognition and immune responses (33). Similarly, the newly identified Dectin-2 N170I mutant was shown to reduce fungal recognition and immune responses (34). Crucially, genetic deficiencies are present prior to the initiation of patient’s primary treatment and immune suppression, thereby offering an early indication of a patient’s susceptibility to IA. Whilst these risk factors have been associated with IA, they are not yet widely applied in clinical practice (22, 33, 35, 40). Using pre-emptive risk factors to stratify patients has significant promise and has recently been tested. Mutations in Dectin-1 and DC-SIGN, respiratory viral infection, allogeneic stem cell transplant and *Aspergillus* PCR positivity were used to stratify patients in a predictive disease model. Patients with no risk factors had a 2.4% probability of developing IA whilst patients with four or more risk factors had a 79% probability of developing IA (22). A future study combining the risk factors described by White P. L. et al. and those described in this study may further enhance patient stratification.

In this study, we identified two CLR mutations in our patient cohort and investigated each patient’s CLR expression before associating these factors with the incidence of IA. The S32G mutation identified in Mcl has been previously described (rs4304840). This missense mutation results in the substitution of serine to glycine at position 32; this is not thought to have functional consequence. In agreement with this, we identified no association between the incidence of Mcl S32G and IA. Our study also identified a novel N170I mutation in Dectin-2. This mutation resulted in an early stop codon located in the carbohydrate binding domain of the CLR. We recently characterized this mutation and showed that it results in reduced receptor expression and deficient anti-fungal immune responses (34). This mutation was identified in a patient who developed IA and died; however, as this is only one patient the statistical association of this mutation and IA cannot be undertaken.

We determined that high (above the median in this study) Mcl and Dectin-1 expression in SCT patients and low (below the median in this study) Dectin-1 expression in AML patients may be associated with an increased risk of IA. Dectin-1 has been extensively shown to drive protective immune responses against fungal pathogens including *Aspergillus* (28, 41, 42). Therefore, as was found with the Dectin-1 Y238X mutant, it is unsurprising the low/deficient Dectin-1 expression in AML patients may increase IA susceptibility. However, it is not clear why high Dectin-1 expression in SCT patients may be associated with an increased risk of IA. There is limited research describing the functional role of Mcl. Mcl-deficient mice produced defective immune responses against TDM (mycobacterial trehalose dimycolate) (43). A collaborative role for Mcl and the Mincle heterodimerising and enhancing the recognition of carbohydrate and lipids molecules has been identified; however, the functional consequences of this heterodimer CLR complex remain largely unknown (44–46). An explanation for the high Mcl expression identified in SCT patients who developed IA is not clear and requires further investigation.

### Functional Response and IA

Previous studies aiming to describe risk factors associated with IA have not investigated patient’s functional response against inflammatory stimuli or *Aspergillus*. Patient PBMCs have been used to identify those most receptive to immunotherapy with positive results correlating assay outcome and patient outcome. However, these investigations were completed retrospectively after all patients had received treatment (47, 48). Here, we investigated whether patient’s functional immune response could be used to predict IA incidence. Our research describes how the results from a simple assay can be used as a novel IA risk factor that could drive a personalised medicine approach.

In this study we describe how LPS- and *Aspergillus*-induced TNF and IL-6 response can be used to identify the SCT patients most susceptible to IA. PBMCs produce pro-inflammatory cytokines and drive protective immune responses when challenged with *Aspergillus*-extracted chitin or live *Aspergillus* (49, 50). The cytokines IL-6 and TNF play a key role in anti-*Aspergillus* immunity with mice deficient in either cytokine possessing an increased susceptibility to Aspergillosis (37, 38). In agreement, the patients in this study who were unable to produce TNF and/or IL-6 against *Aspergillus* possessed enhanced susceptibility to IA. Similar results were attained with *Candida albicans* where fungal disease was associated with a delayed secretion of cytokines from myeloid cells and T cells (51).

Our functional assay results determined that patients able to produce an LPS-induced response but lacking an *Aspergillus*-induced response possessed the highest IA susceptibility. Anti-*Aspergillus* immune responses are complicated and require collaboration between numerous receptors, signalling molecules and cell types to produce a protective immune response. Patients with haematological malignancies often possess immune defects and have highly variable immune cell counts (52). Here, our functional assay may be able to discriminate between patients with high disruption to their anti-fungal immune response and those with minimal disruption. It is likely those with high disruption possessed deficiencies in anti-fungal immune components that we did not screen for. In contrast to the *Aspergillus*-induced response, the majority of patients were able to produce LPS-induced TNF and/or IL-6. The LPS-induced response requires only TLR4 and CD14 signalling to produce a robust pro-inflammatory response (53) and PBMCs produce large quantities of TNF and IL-6 within 4 h of LPS stimulation (54). Crucially, TLR signalling is retained in patients with a haematological malignancy even after SCT, radiotherapy or chemotherapy and is often responsible for graft versus host disease, gut toxicity and chronic pain (55–57). This maintenance of TLR signalling likely explains why most patients in this study produced LPS-induced responses.

## Conclusion

Our research is the first to stratify patients at high risk of fungal disease according to their functional anti-*Aspergillus* immune responses and their CLR status. We describe novel risk factors including patient’s LPS- and *Aspergillus*-induced TNF and IL-6 PBMC response, and the increased stratification that can be achieved through combining patient’s functional responses with their CLR expression levels. We also identified two CLR mutants of which Mcl S32G did not influence IA susceptibility, whilst Dectin-2 N170I likely does increase IA susceptibility. These risk factors were associated with the incidence of IA within our study and resulted in patient stratification into three cohorts. Crucially, within our small patient cohort, we were able to stratify patients into a 0% risk group (those with an *Aspergillus*-induced TNF and/or IL-6 response), this represents an important step promoting a personalised medicine approach where this cohort’s primary disease therapy is prioritised. We were also able to identify a high-risk cohort (those with an LPS- but not *Aspergillus*-induced TNF and/or IL-6 response and high Mcl and/or Dectin-1 expression), this highly susceptible cohort should have a personalised medicine approach that considers their IA susceptibility. Whilst our research was a pilot study that requires further validation in a larger study, we describe novel risk factors and a novel strategy that promotes a personalised medicine approach to haematology patient’s fungal disease.

## Data Availability Statement

The raw data supporting the conclusions of this article will be made available by the authors, without undue reservation.

## Ethics Statement

The studies involving human participants were reviewed and approved by Health and Care Research Wales. The patients/participants provided their written informed consent to participate in this study.

## Author Contributions

SO, PT, and RB contributed to conception and design of the study. JG undertook experiments and wrote the manuscript. AT, DF, and RP undertook experiments. PW anonymised and provided patient samples and data, and supported study analysis. WI and KW recruited patients and took samples. All authors contributed to manuscript revision, read, and approved the submitted version.

## Funding

SO was funded by a Sir Henry Dale Fellowship jointly funded by the Wellcome Trust and the Royal Society (grant number 099953/Z/12/Z) and by the T. Maelgwyn Davies Bequest fund. PT is supported by a Wellcome Trust Investigator Award (107964/Z/15/Z) and the United Kingdom Dementia Research Institute.

## Conflict of Interest

The authors declare that the research was conducted in the absence of any commercial or financial relationships that could be construed as a potential conflict of interest.

## Publisher’s Note

All claims expressed in this article are solely those of the authors and do not necessarily represent those of their affiliated organizations, or those of the publisher, the editors and the reviewers. Any product that may be evaluated in this article, or claim that may be made by its manufacturer, is not guaranteed or endorsed by the publisher.
