# Supplementary material for: A Novel Strategy to Identify Haematology Patients at High Risk of Developing Aspergillosis
Source: Front Immunol. 2021 Dec 16;12:780160. doi: 10.3389/fimmu.2021.780160 (PMC8716727; doi:10.3389/fimmu.2021.780160)
Supplement: Supplementary file 1 [file DataSheet_1.docx]

**A Novel Strategy to Identify Haematology Patients at High Risk of Developing Aspergillosis**

James S. Griffiths^1,2^, P. Lewis White^,3^, Aiysha Thompson^1,4^, Diogo M. da Fonseca^1,5^, Robert J. Pickering^1,6^, Wendy Ingram^7^, Keith Wilson^7^, Rosemary Barnes^1^, Philip R. Taylor^1,4^, Selinda J. Orr^1,5,*^.

**Supplementary Material**

**Supplementary Table 1:** Primers and probes used in this study

| **qPCR Probe Target** | **Product** | **Company** |
| --- | --- | --- |
| HPRT | 02800695 m1 | ThermoFisher |
| *CLEC7A* (Dectin-1) | 00224028 m1 | ThermoFisher |
| *CLEC6A* (Dectin-2) | 01073951 m1 | ThermoFisher |
| *CLEC4D* (Mcl) | 01073582 m1 | ThermoFisher |
| *CLEC4E* (Mincle) | 00372017 m1 | ThermoFisher |
|  | | |
| **Target** | **Primer Binding** | **Sequence** |
| Dectin-1 | Forward 1 (5’-3’) | atggaatatcatcctgatttagaaa |
|  | Forward 2 (5’-3’) | Ttcagcatgtcactaaattcc |
|  | Reverse 1 (3’-5’) | Ttacattgaaaacttcttctcacaa |
|  | Reverse 2 (3’-5’) | aggagattagagcccagttg |
| Dectin-2 | Forward 1 (5’-3’) | atgatgcaagagcagcaa |
|  | Forward 2 (5’-3’) | cccagcttcttggaagtca |
|  | Reverse 1 (3’-5’) | tcataggtaaatcttattcatctcacat |
|  | Reverse 2 (3’-5’) | actcattcagctgctggac |
| Mcl | Forward 1 (5’-3’) | atggggctagaaaaacctca |
|  | Forward 2 (5’-3’) | acaagacgtgggctgaga |
|  | Reverse 1 (3’-5’) | ctagttcaatgttgttccaggtattt |
|  | Reverse 2 (3’-5’) | ccactgacctttggcatt |
| Mincle | Forward 1 (5’-3’) | atgaattcatctaaatcatctgaaac |
|  | Forward 2 (5’-3’) | accatttcctgggcgtta |
|  | Reverse 1 (3’-5’) | ttaaagagattttcctttgttcaaa |
|  | Reverse 2 (3’-5’) | tcctgctcctcctgtgag |

**Supplementary Table 2:** No association between IA and mortality, and functional immune response and mortality. This data was produced from a contingency multivariate statistical analysis of IA incidence against incidence of mortality and PBMC anti-LPS and anti-*Aspergillus* cytokine response against incidence of mortality. Fisher’s exact test was used to identify statistical significance. Where two variables were examined, statistical significance was set at **p*<0.05; significant values are highlighted bold.

| **Patient Group** | **Parameter** | **Mortality** | **Survival** | **Odds Ratio** | **95% CI** | **p Value** |
| --- | --- | --- | --- | --- | --- | --- |
| Total (40) | IA | 4/15 | 6/25 | 1.152 | 0.26 to 4.99 | 1 |
| AML (17) | IA | 1/6 | 3/11 | 0.533 | 0.04 to 6.65 | 1 |
| SCT (23) | IA | 3/9 | 3/14 | 1.833 | 0.27 to 12.07 | 0.643 |
|  |  |  |  |  |  |  |
| Total (40) | No *Aspergillus* TNF response | 5/15 | 14/25 | 0.392 | 0.10 to 1.49 | 0.204 |
| Total (40) | No *Aspergillus* IL-6 response | 6/15 | 13/25 | 0.615 | 0.16 to 2.25 | 0.526 |
| SCT (23) | No *Aspergillus* TNF response | 3/9 | 8/14 | 0.375 | 0.06 to 2.15 | 0.40 |
| SCT (23) | No *Aspergillus* IL-6 response | 3/10 | 8/13 | 0.268 | 0.046 to 1.54 | 0.214 |
